# Moyamoya Disease in a Child With Fanconi Anemia: An Anomaly or a Complication

**DOI:** 10.7759/cureus.54455

**Published:** 2024-02-19

**Authors:** Samin Alavi, Mitra Khalili, Zahra Khaffafpour, Negar Shams

**Affiliations:** 1 Hematology/Oncology, Pediatric Congenital Hematologic Disorders Research Center, Research Institute for Children's Health, Shahid Beheshti University of Medical Sciences, Tehran, IRN; 2 Pediatric Radiology, Shahid Beheshti University of Medical Sciences, Mofid Children’s Hospital, Tehran, IRN; 3 Pediatric Hematology/Oncology, Pediatric Congenital Hematologic Disorders Research Center, Research Institute for Children's Health, Shahid Beheshti University of Medical Sciences, Tehran, IRN; 4 General Practice, Pediatric Congenital Hematologic Disorders Research Center, Research Institute for Children's Health, Shahid Beheshti University of Medical Sciences, Tehran, IRN

**Keywords:** bilateral disease, mri, internal carotid artery, moyamoya disease, fanconi anemia

## Abstract

Fanconi anemia (FA) is an inherited bone marrow failure syndrome associated with congenital anomalies and a predisposition to cancer. We report the case of a 9-year-old boy with FA who developed an abrupt onset of hemiplegia and dysarthria. The diagnosis of moyamoya disease (MMD) was suggested by magnetic resonance angiography (MRA) which demonstrated severe stenosis in the right internal carotid artery along with collateral vessel formation in the right basal ganglia. It is questioned whether the moyamoya pattern in this case is part of congenital malformations associated with FA or is the result of recurrent bleedings around the carotid siphon.

## Introduction

Fanconi anemia (FA) is a heterogeneous autosomal recessive disorder characterized by congenital malformations, progressive marrow failure, and predisposition to acute myelogenous leukemia and other malignancies. Congenital anomalies are variable and may involve the skeletal system as well as other organ systems including ocular, auditory, renal, genital, and central nervous systems [1,2]. Congenital central nervous system abnormalities in children with FA are poorly characterized.

Moyamoya disease (MMD) is an uncommon disease of the central nervous system. It was first reported in Japan in 1957. The word “Moyamoya” in Japanese means “a puff of smoke” referring to the characteristic angiographic appearance of the collateral circulation in the brain. MMD is rarely associated with FA [3]. It results from chronic stenosis or occlusion of the distal carotid artery and subsequent formation of collateral vessel networks. One possible etiology in the development of MMD in FA is recurrent bleeding around the carotid siphon resulting in progressive narrowing of the carotid artery [4]. Considering that FA is associated with multiple congenital anomalies, narrowing of the carotid artery could be considered a congenital defect [5].

## Case presentation

A 10-year-old boy with FA born to consanguineous parents was admitted to the emergency department with sudden onset of slurred speech, left-sided weakness, and perioral paresthesia. It was not associated with fever, convulsion, or loss of consciousness. There was no history of head trauma. The parents complained of similar transient attacks during the last three months which had resolved spontaneously without any medical evaluation.

**Figure 1:**
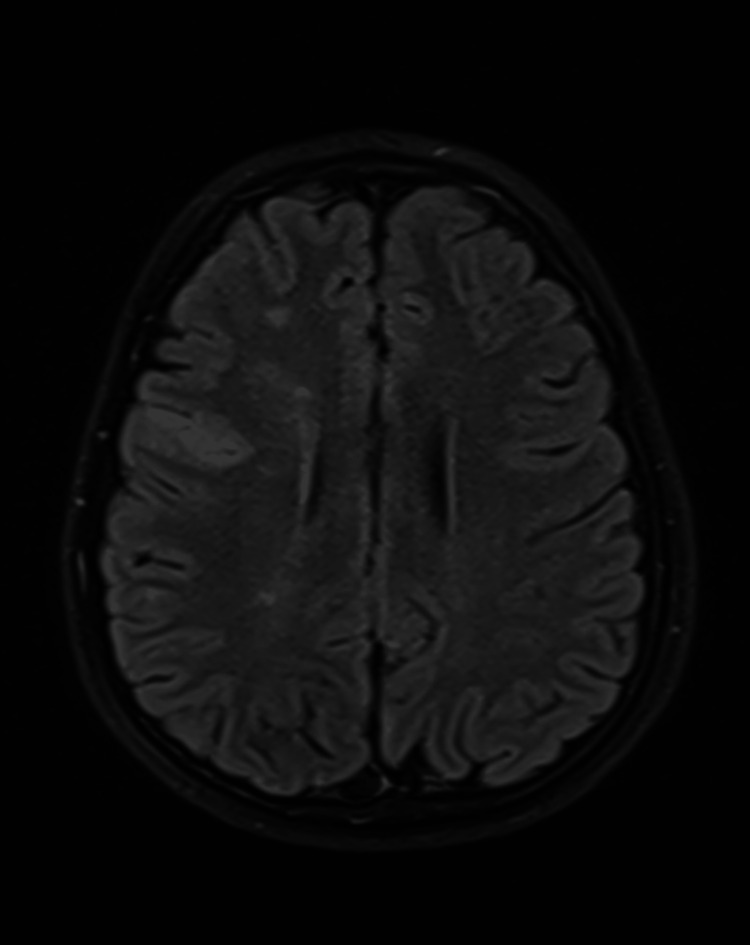
MRI, Axial FLAIR image showing abnormal high signal in the right frontal lobe cortex and also in the centrum semiovale in the deep watershed area.

**Figure 2:**
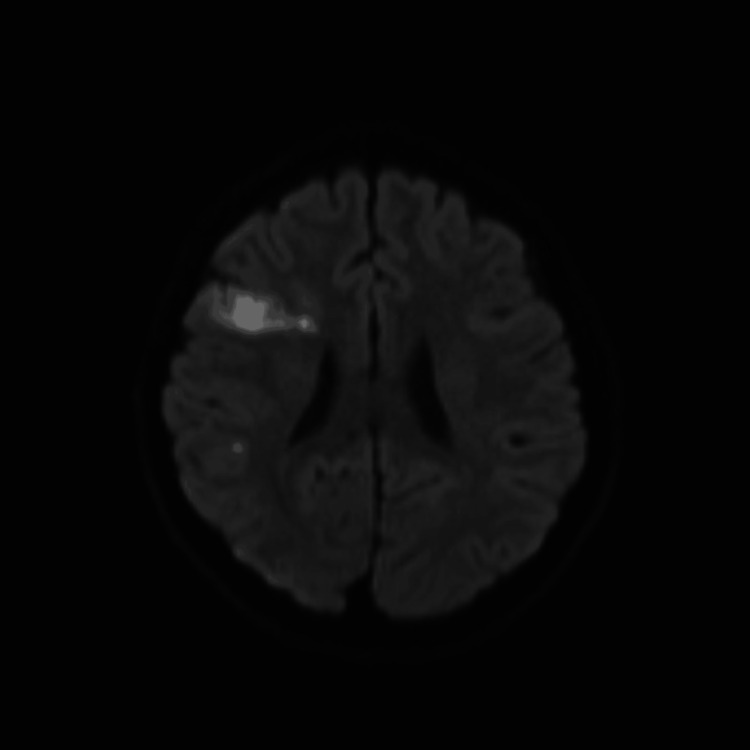
MRI, Axial DWI image showing diffusion restriction in the right frontal lobe in favor of ischemia.

**Figure 3:**
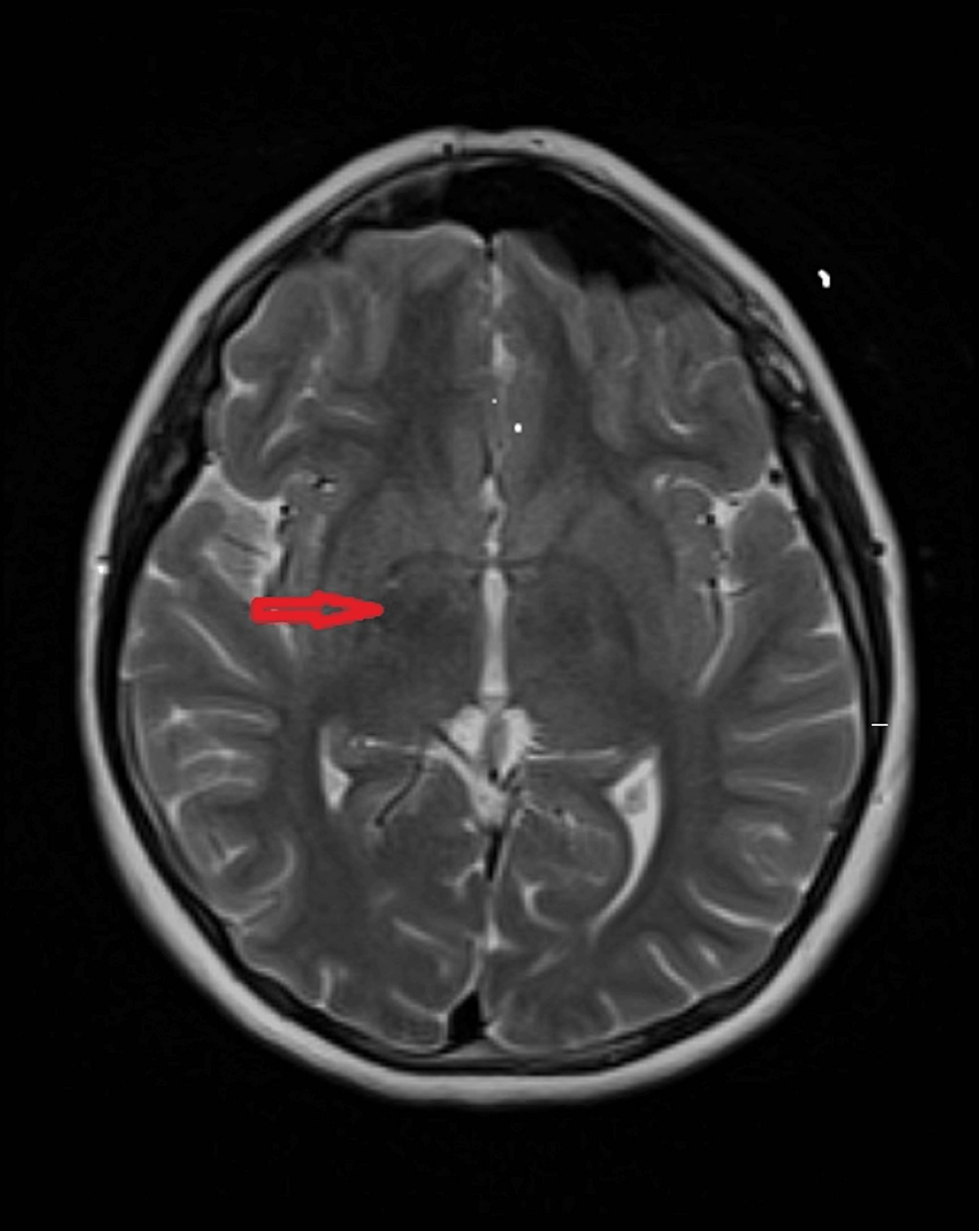
MRI, T2 weighted axial images showing faint collateral vessels (brush sign) in the right basal ganglia (red arrow).

**Figure 4:**
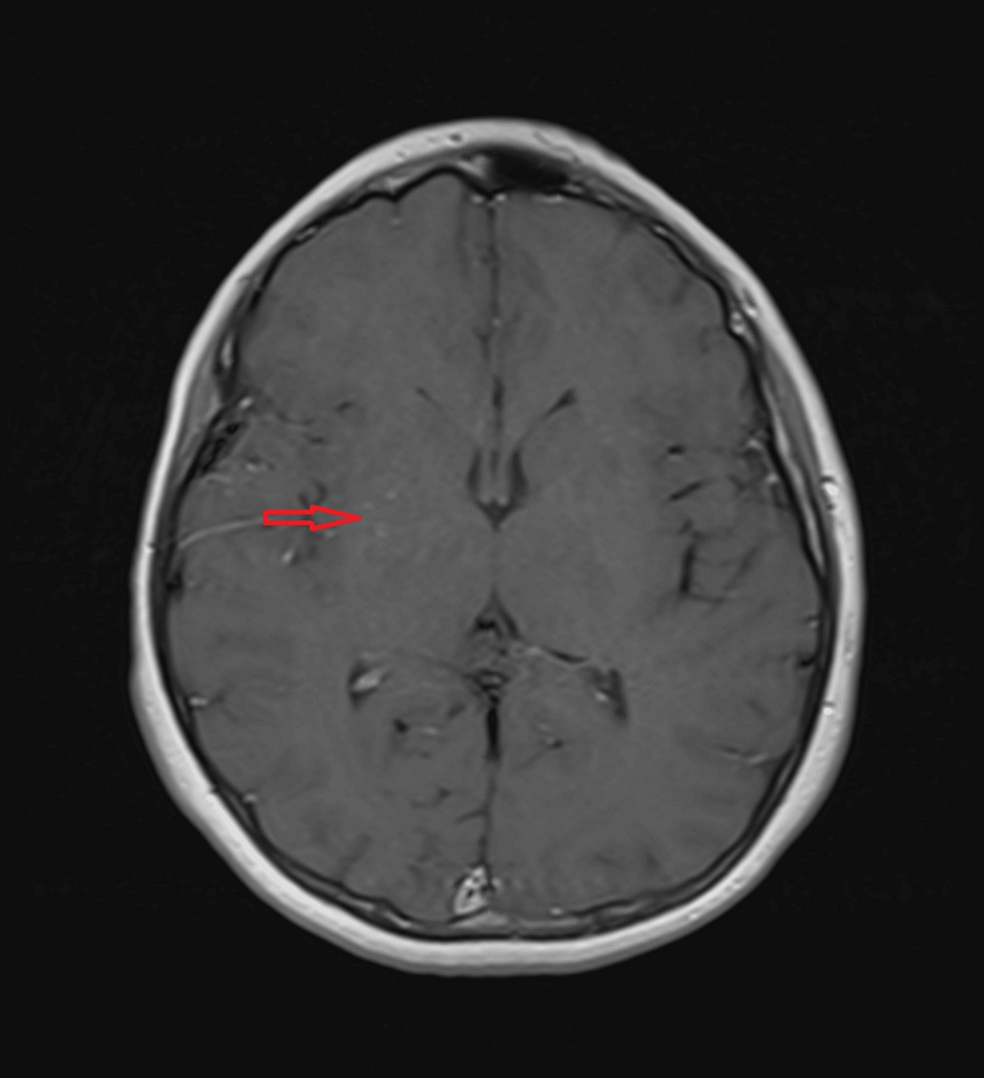
MRI, contrast-enhanced T1 weighted axial image showing faint collateral vessels (brush sign) in the right basal ganglia (red arrow)

**Figure 5:**
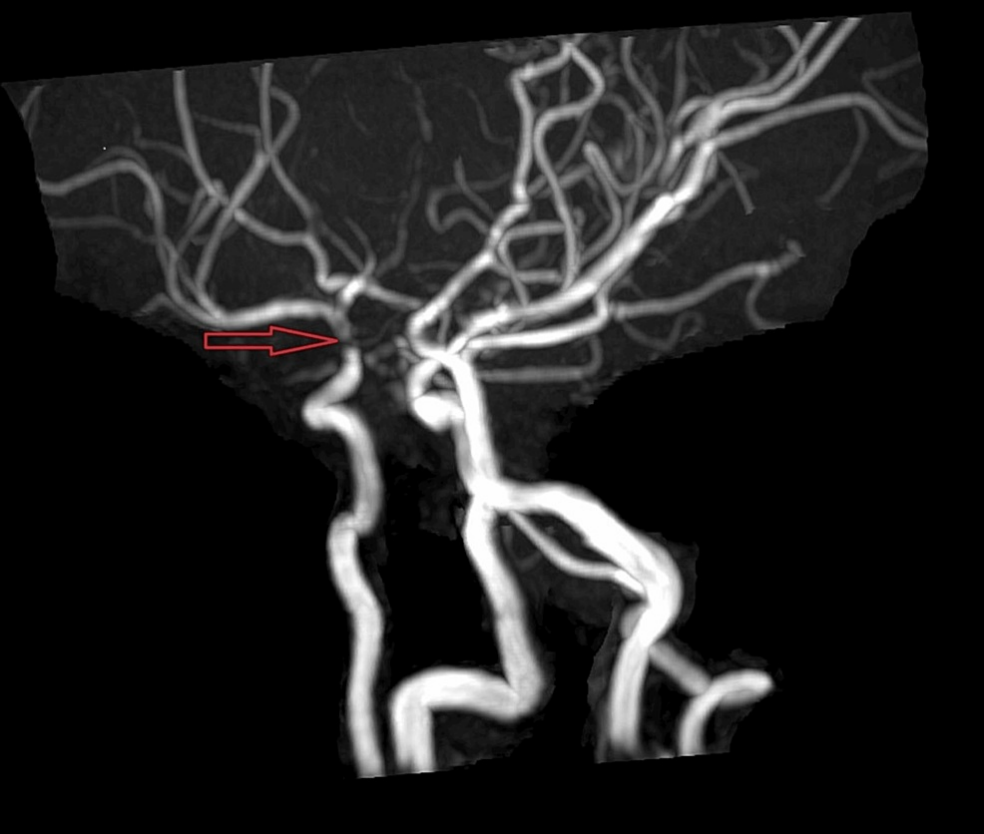
MRA showing severe stenosis in the supraclinoid portion of the right internal carotid artery with filling of the circle of Willis from the contralateral left carotid artery.

Physical examination revealed left hemiparesis of upper and lower limbs associated with facial drooping upon arrival. He was transferred to the intensive care unit and recovered spontaneously after a few hours without any residual weakness. The patient had been diagnosed with FA at the age of eight. He was on oxymetholone (5 mg/kg) and eltrombopag (50 mg) daily. Due to severe thrombocytopenia, a brain CT scan was performed which was not diagnostic for intracranial hemorrhage. According to the neurological symptoms, magnetic resonance imaging (MRI) was ordered which showed a wedge-shaped area of restricted diffusion along the right frontal lobe with high signal foci in the right centrum semiovale in favor of the anterior watershed infarct (Figures 1-2). T2 weighted and contrast-enhanced T1 weighted axial images showed faint collateral vessels (brush sign) in the right basal ganglia (Figures 3-4). Magnetic resonance angiography (MRA) depicted severe stenosis in the supraclinoid portion of the right internal carotid artery with the filling of the circle of Willis through the contralateral artery (Figure 5).

The flow was normal in the petrous and cavernous parts of the bilateral internal carotid arteries. MMD was suggested for the patient. The patient was a candidate for revascularization surgery due to repeated ministroke attacks; however, it was not practical due to severe thrombocytopenia, which could act as a protective factor for the prevention of a stroke in the patient.

## Discussion

FA is the prototype of congenital anomalies involving almost all organ systems. A research center for FA patients has published its experience on central nervous system lesions of patients with FA where cranial MRIs of 34 patients with FA were retrospectively evaluated. At least one pathological finding was demonstrated in 22 (65%) patients. These findings included the corpus callosum and other supratentorial malformations, pituitary, craniovertebral junction and posterior fossa abnormalities, vascular lesions, and intracerebral calcifications [6].

Cerebrovascular accidents occurring in FA are generally hemorrhagic due to severe thrombocytopenia; however, the possibility of an underlying vascular anomaly should also be considered. Association of FA with cerebrovascular anomalies is a rare phenomenon. A 12-year-old boy from India has been reported with hypoplasia of basilar, posterior cerebral, and posterior communicating arteries [7]. A 14-year-old boy with FA and agenesis of the internal carotid artery has been reported whose MRI showed hyperintensities in the occipital lobe and cingulate gyrus associated with the absence of the left carotid channel. The patient was asymptomatic for many years owing to the rich collateral network of vessels from the circle of Willis [5].

Recently, the Research Committee on MMD has described the new “Diagnostic Criteria 2021” for MMD to update the definition of the disease, diagnostic imaging, and the concept of quasi-MMD (moyamoya syndrome) [8]. Although, the diagnostic criteria in 2015 had removed the distinction between “definite case” and “probable case” and emphasized that there is nevermore any need to distinguish between unilateral and bilateral moyamoya cases, the “Diagnostic Criteria 2021” clearly declared both unilateral and bilateral cases as MMD. Accordingly, a stenotic lesion at the terminal portion of the internal carotid artery is the most fundamental feature of MMD. On MRI and MRA, stenosis or occlusion of the terminal portion of the intracranial internal carotid artery, decrease in the outer diameter of the terminal portion of the intracranial internal carotid and the horizontal portion of the middle cerebral artery bilaterally on heavy T2-weighted MRI, and abnormal vascular networks in the basal ganglia and/or periventricular white matter are required for diagnosis of MMD. In the case of cerebral angiography, both unilateral and bilateral cases can be diagnosed as MMD [8]. The present case showed occlusion of the intracranial portion of the internal carotid artery and the horizontal portion of the middle cerebral artery along with abnormal vascular networks in the basal ganglia and/or periventricular white matter. The criteria of decrease in the outer diameter of the terminal portion of the internal carotid is needed for differentiation of atherosclerosis from MMD, and since atherosclerosis is not expected to exist in children, heavy T2-weighted MRI was not performed for the current case.

MMD has been associated with various systemic diseases. Sickle cell anemia, FA, iron deficiency anemia, hereditary spherocytosis, homocystinuria, and essential thrombocythemia are among the hematologic disorders that are very rarely complicated with MMD [3,9]. The association with hemoglobinopathies is less frequently observed. The present case was a unique case who developed two episodes of transient stroke recovering spontaneously without any intervention.

A 10-year-old girl with FA has been reported from Saudi Arabia presenting with a sudden onset of right-sided weakness without facial paresis. Brain MRI showed an area of acute ischemic infarct in the left frontal lobe and an old infarction in the right parietal periventricular white matter with multiple small lacunar infarcts. The brain MRA showed a narrow distal internal carotid artery with a "puff of smoke" sign on the right side suggestive of MMD [4]. The appearance of "puff of smoke" was not evident in the current case; however, as we mentioned, it is no longer a diagnostic criterion for MMD. Likewise, a 10-year-old boy with FA was admitted because of acute hemiplegia of the left side. Internal carotid arteriography disclosed a moyamoya cerebrovascular pattern on the right side. Although MMD may be acquired, the authors have suggested it as an associated congenital anomaly of patients with FA [10].

In a clinical database of 434 patients, two patients with FA and MMD have been reported. Both cases belonged to the FA complementation group C (FACC) [11]. We assume that moyamoya anomaly may be related to a specific type or combination of mutations in FA.

Ring finger protein 213 (RNF213), also known as Mysterin, is a known susceptibility gene for the development of moyamoya arteriopathy [12]. Although several polymorphisms for the RNF213 gene have been reported, variants of this gene have not been identified to date in FA patients [13].

Treatment in MMD is aimed at reducing the risks of both ischemic and hemorrhagic stroke. There are no investigations to guide definitely on preventive therapy for the ischemic subtype. Antithrombotics are not recommended due to concerns for increased risk of hemorrhage and lack of efficacy for prevention of ischemic events. Existing data suggest antiplatelet drugs mainly in Western countries, where the ischemic subtype of MMD is more common [14]. We referred the patient for revascularization surgery, but due to severe thrombocytopenia, the procedure was not approved, and the low platelet counts were considered as a protective factor against further strokes.

## Conclusions

This was a very rare case of MMD in a child with FA considering its transient, self-limiting course of hemiparesis with spontaneous clinical recovery. The authors recommend that MRI and MRA should be performed in every patient with FA despite the lack of any symptoms. Furthermore, genetic mutation analysis for FA complementation groups and Ring Finger Protein 213 is suggested to explore any possible association between the two conditions.
